# Cuprous oxide nanoparticles inhibit the growth of cervical carcinoma by inducing autophagy

**DOI:** 10.18632/oncotarget.17854

**Published:** 2017-05-15

**Authors:** Leilei Xia, Ye Wang, Ya Chen, Jiuqiong Yan, Fan Hao, Xiaoling Su, Caihong Zhang, Mingjuan Xu

**Affiliations:** ^1^ Department of Obstetrics and Gynaecology, Changhai Hospital, The Second Military Medical University, Shanghai 200433, P.R. China; ^2^ Department of Urology, Changhai Hospital, The Second Military Medical University, Shanghai 200433, P.R. China; ^3^ Department of Obstetrics and Gynecology, Shanghai Tenth People’s Hospital, Tongji University School of Medicine, Shanghai 200072, P.R. China

**Keywords:** cuprous oxide nanoparticle, cervical cancer, nanomedicine, therapy, AKT/mTOR pathway

## Abstract

Cervical carcinoma is one of the main causes of women’s cancer, and substantial side effects from standard treatment including platinum-based chemotherapy limit the options for escalation. In this paper, using cervical cancer cell lines and tumor-bearing mice as models, we report that CONPs could inhibit the proliferation of cancer cells *in vitro* and *in vivo*. Especially CONPs could inhibit tumor growth as cisplatin without weight loss. CONPs could also induce autophagy through AKT/mTOR pathway, which demonstrates that CONPs has the potential clinical applications.

## INTRODUCTION

Cervical carcinoma has the highest incidence in gynaecological malignancy and is one of the main causes of cancer-related deaths in females [1], especially in the developing and undeveloped countries [1–4]. Although the incidence and mortality rates have decreased due to the wide use of neoadjuvant radio and chemotherapy [5] that can greatly improve the outcome of cervical carcinoma in a group of patients, intrinsic and acquired resistance of the neoplastic cells to the therapy still occurs in patients. In addition, substantial side effects from standard treatment including platinum-based chemotherapy limit the options for escalation, which is a major clinical challenge for the therapy.

As nanotechnology progresses, nanomedicine, the combination of nanotechnology and biomedical, has brought improving future in cancer treatment [6–8]. The idea that nanomedicine may induce autophagy in cancer cells has attracted mounting attention as it could be a useful new approach to treat cancer [9–11]. Inspired by the arsenic trioxide and Artemisia annua L, the traditional Chinese remedies, which have been proved to cure APL (acute promyelocytic leukemia) and malaria effectively [12], our group combined the nanomedicine and the traditional Chinese medicine and found that CONPs had ideal potential pharmacological effects on tumor therapy with little toxic effects [13, 14].

In this study, we performed the cytotoxicity of CONPs on cervical carcinoma cell lines and the therapeutic effects on cervical carcinoma xenograft in nude mice to better evaluate the effects of CONPs on cervical carcinoma therapy. We found that CONPs could strongly inhibit growth and induce apoptosis in both cultured cells and tumor bearing mice. Furthermore, the findings revealed that CONPs could activate the autophagy and Akt/mTOR pathways in cervical cancer cells.

## RESULTS

### CONPs inhibit the growth and the migration of cervical cancer

**Figure 1:**
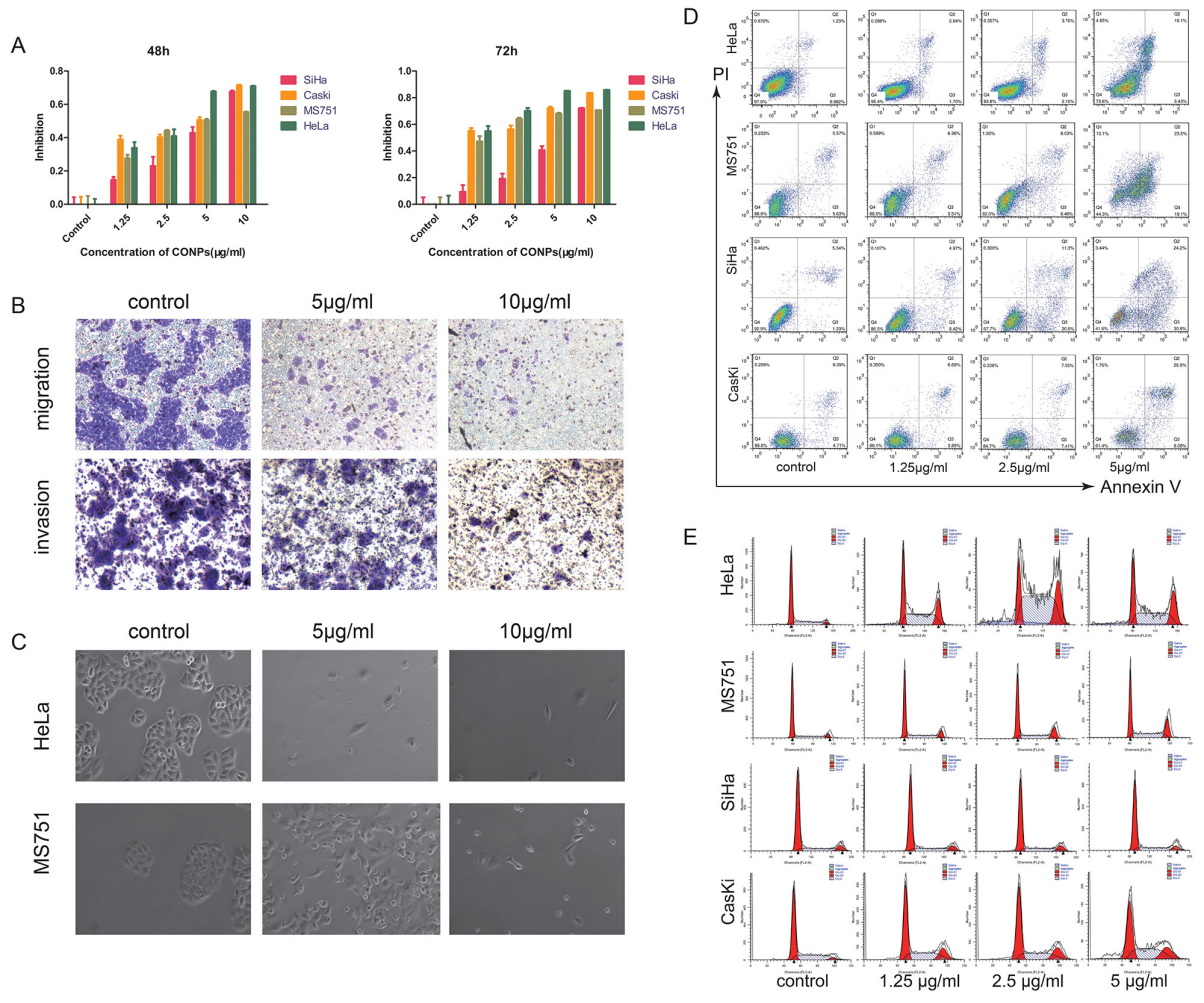
CONPs induce apoptosis and inhibit the migration of cervical carcinoma **(A)** CCK8 assay was performed to access the cytotoxicity of CONPs on the proliferation of cervical cancer cell lines (SiHa, Caski, MS751, HeLa). A summary of experiments measuring cell death at 48, and 72 h is shown. The viability of the cells was reduced by CONPs in a dose-and time-dependent manner. **(B)** MS751 were treated with various concentration of CONPs for 24h. Top, transwell migration assays; bottom, transwell invasion assays. **(C)** MS751 and HeLa treated with various concentration of CONPs for 48h were photographed by electron microscopy. The normal cells are in cobblestone appearance, while the dead cells are spherical. **(D)** Apoptosis assay of these cells incubated with various concentrations of CONPs for 48 hours. CONPs induced apoptosis of several cervical cancer cells significantly at the concentration of 2.5 µg/mL and 5 µg/mL for 48 hours. Cells in the early stage of apoptosis are in the lower right quadrant, cells in the late stage of apoptosis are in the upper right quadrant, necrotic cells are in the upper left quadrant. **(E)** Cell cycle progression assays were performed on the cells treated with various concentrations of CONPs for 48 hours. After 48 hours, CONPs blocked cell cycle at G0/ G1 phase.

The synthetic method, main characteristics of CONPs are introduced in our previous study [13, 14]. Cell viability was detected by the Cell Counting Kit-8 assay. The data indicated that CONPs could inhibit the proliferation of cervical cancer cells in a time and concentration dependent manner. After the treatment of CONPs for 48h, our data showed that the half-maximal inhibitory concentration for HeLa cells, MS751 cells, CasKi cells and SiHa cells were 3.04 μg/ml, 5.27 μg/ml, 3.48 μg/ml and 5.76 μg/ml respectively (Figure 1A). Also, the invasiveness of cervical cancer cells was detected by the transwell assays. The results indicated that with the increase of the concentration of CONPs, the migration and invasion of MS751 cells were reduced markedly (Figure 1B). The morphology of cells treated with CONPs have changed greatly. The results showed that cells in control group were cobblestone and cells treated with CONPs were floating with a spherical shape while there is no difference between the living cells and the control group. The effects also were concentration dependent (Figure 1C). In addition, the apoptosis assays were conducted on cervical cancer cells. The data indicated that CONPs can evidently induce cells apoptosis in a concentration-dependent pattern (Figure 1D). Cell cycle were evaluated by fluorescence activated cell scan(FACS). The detection results indicated that cell cycle was blocked in G1/G0 by CONPs in a concentration dependent manner (Figure 1E).

### CONPs suppress the growth of cervical cancer in nude mice

**Figure 2:**
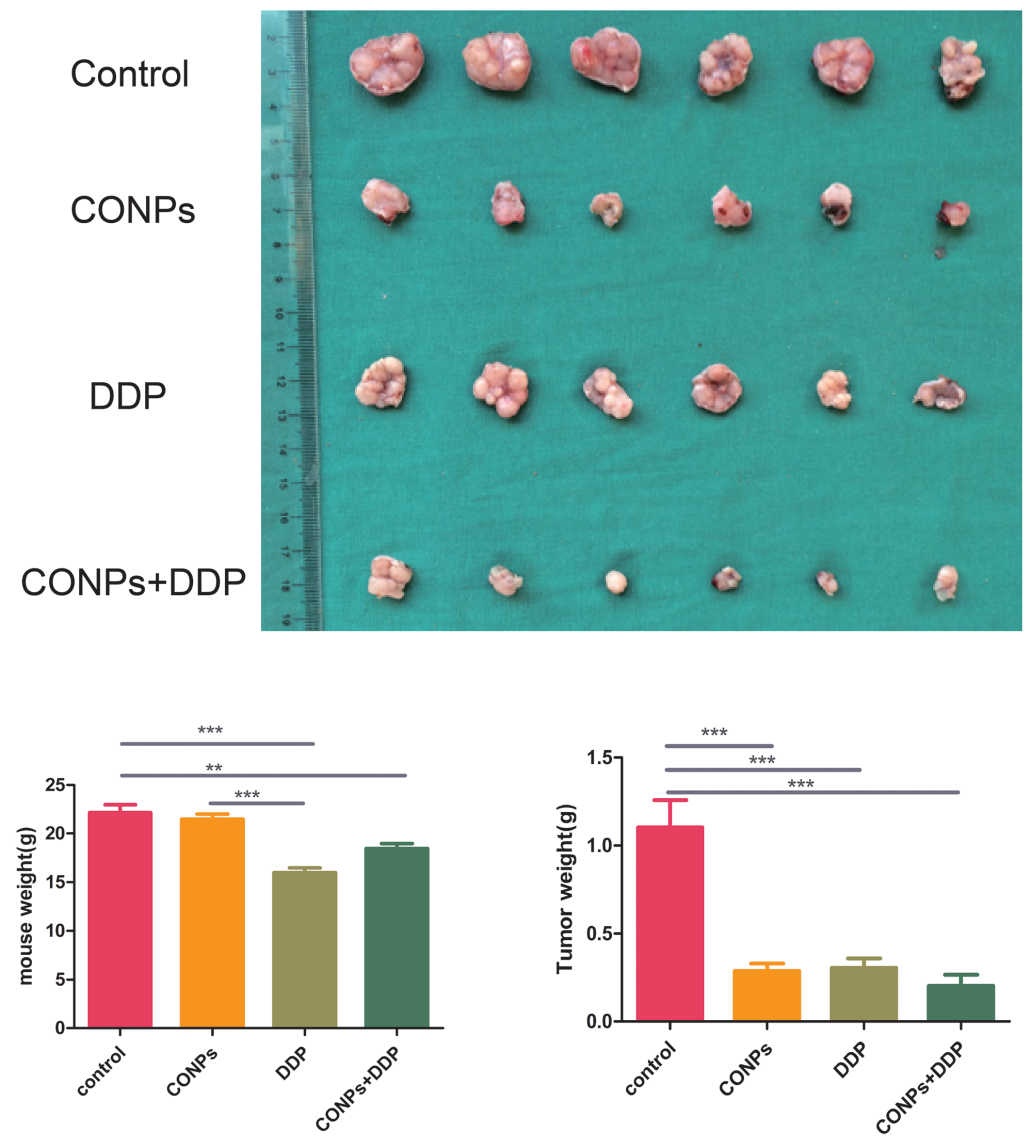
Anti-tumor effects of CONPs Representative pictures of the tumors in the different groups. The diameters of the tumors in the CONPs group were equal to those in the DDP group while distinctly smaller than those in the glucose group. The diameters of CONPs+DDP were the smallest. The weight of the cisplatin group was lower than that of the control group, while there was no difference in weight between the CONPs group and the control group.

The cervical cancer nude mouse subcutaneous planting tumor model was established by transplanting MS751 cells behind the groin of nude mice. We use cisplatin to better evaluate the curative effect of CONPs. CONPs and cisplatin were dissolved in a 5% dextrose. Once tumor reached a diameter of 6mm, the mice were divvied into four experiment groups, including 5% glucose solution control group, the CONPs group, the cisplatin group, and both the CONPs and cisplatin group. CONPs were given as daily injections at 4 mg/kg. The cisplatin solution was injected at 2 mg/kg once two days. In the process of administer, the tumor bulk in the CONPs group was smaller than the control group but comparable to the cisplatin group. Interestingly, even there was no difference in tumor volume between the CONPs group and the cisplatin group, the weight of the cisplatin group was lower than that of the control group, while there was no difference in weight between the CONPs group and the control group (Figure 2). These results indicated that the growth of cervical cancer cells was sharply suppressed by CONPs in mice without weight loss, which is one of the typical side effects of cisplatin.

### The location of CONPs

**Figure 3:**
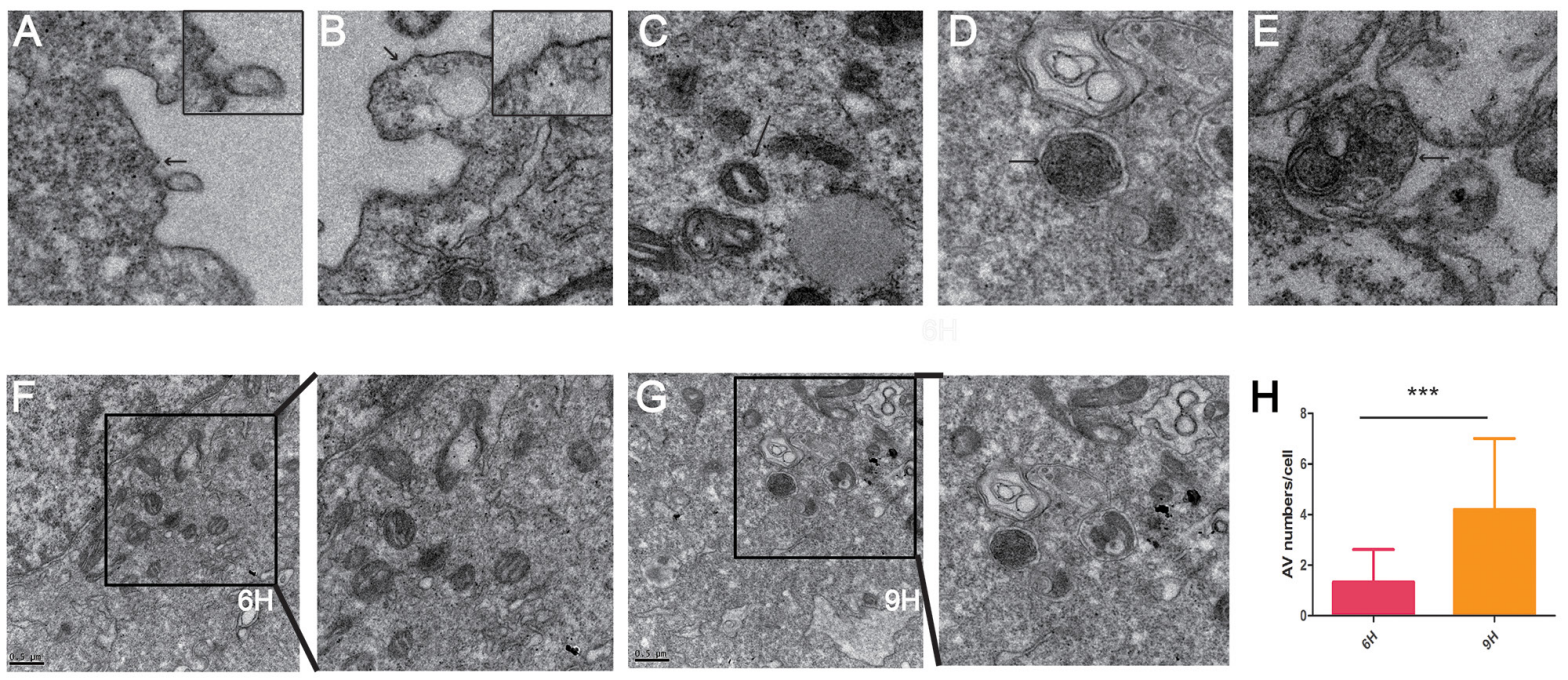
Transmission electron microscope images of MS751 cells treated with CONPs **(A)** CONPs attached to the cytomembrane. **(B)** Slight membrane hollowness containing CONPs. **(C–E)** Intracellular localization of CONPs. **(F-H)** Autophagic vacuole (AV) numbers were increased in a time-depend manner.

To reveal the possible mechanism of the CONPs, it is indispensable to know where the CONPs located in the cells. We performed the transmission electron microscopy assay to better investigate the location of CONPs in MS751 cells treated with 10 µg/ml CONPs. As shown in the Figure 3, we could picture that the CONPs were gathered not only in the cytoplasm but also in the mitochondria and lysosomes. Surprisingly, the longer the treatment, the more the autophagosomes and autophagolysosomes.

### CONPs could damage mitochondrial membrane potential

**Figure 4:**
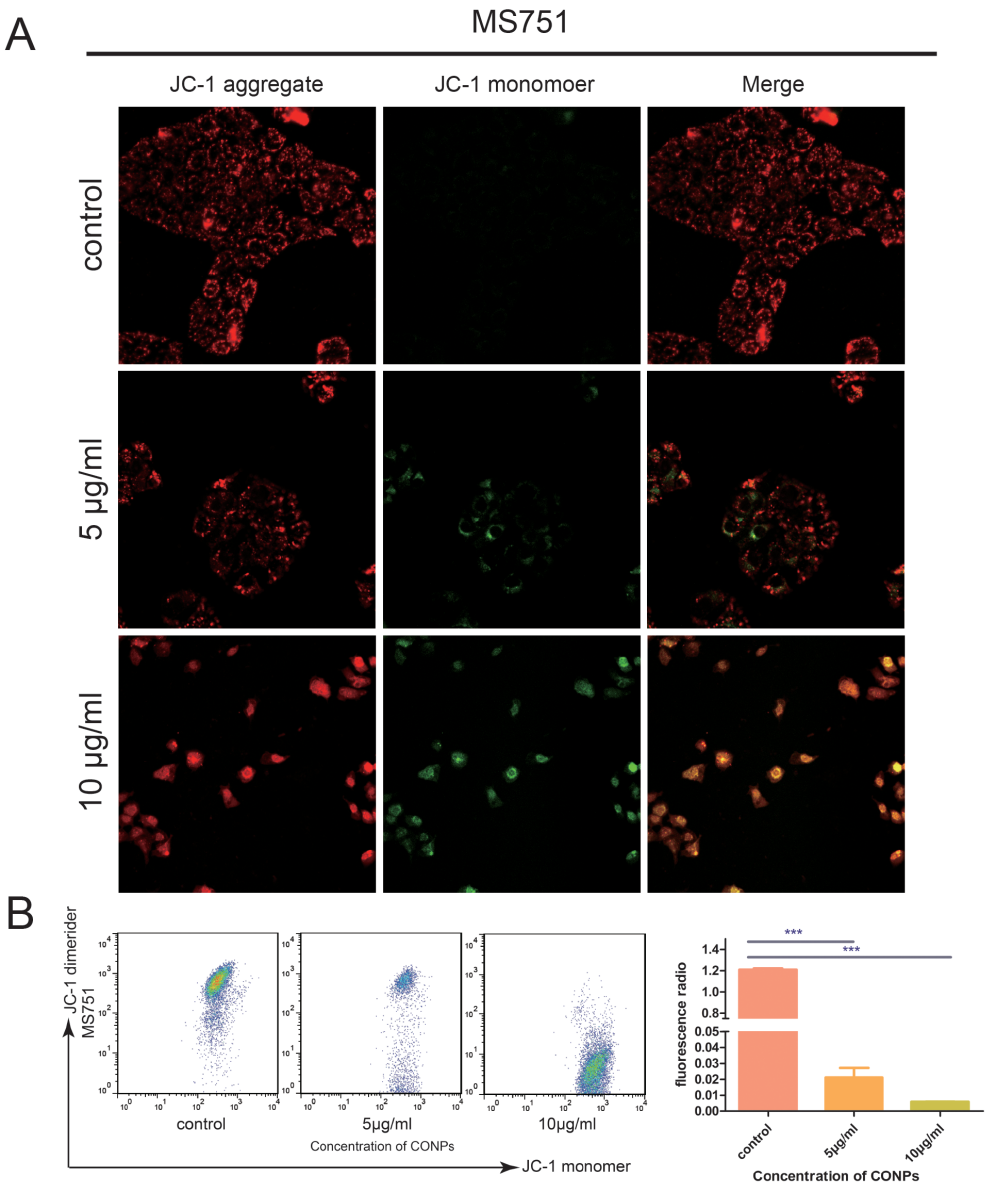
Fluorescence microscopic images of the MS751 cells treated with various concentration of CONPs for 24h **(A)** The red fluorescence intensity was reduced gradually and the green fluorescence intensity was gradually increased when the concentration of CONPs increased. **(B)** The mitochondrial membrane potentials decreased significantly with the treatment of CONPs.

Mitochondria, the key structures of energy production, are involved not only in energy metabolism but also in free radical metabolism. Dysfunction in mitochondria may lead to a globe serious illness [15–17]. We assumed that the CONPs could impair the mitochondria according to the TEM, which showed the CONPs were gathered in the mitochondria. To prove our speculation, mitochondrial membrane potential was detected by confocal laser microscopy and flow cytometry assay. As is shown in Figure 4, with the increase of concentration of CONPs, the green fluorescent intensity strengthened markedly and the red fluorescent intensity weakened. Meanwhile, the cells treated with CONPs were detected by flow cytometry, the analysis were consistent with the results of confocal laser microscopy.

### CONPs may induce autophagy through AKT/mTOR pathway

**Figure 5:**
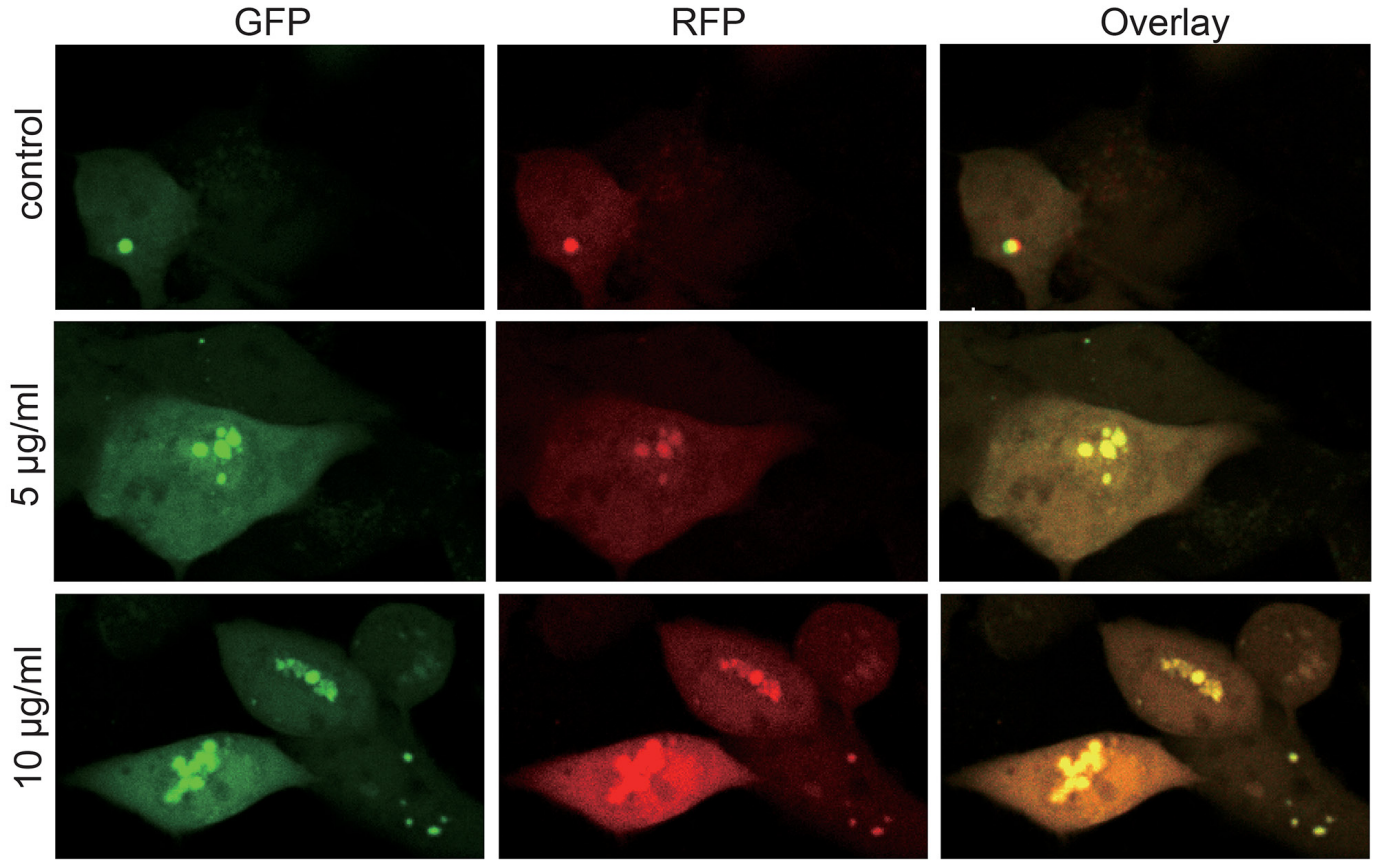
Autophagy flux of the MS751 cells treated with various concentration of CONPs for 72h MS751 treated with adenovirus harboring tandem fluorescent mRFP-GFP-LC3 for 24h were subjected to different concentration of CONPs. Representative pictures of immunofluorescent expressing mRFP-GFP-LC3. GFP dots are green, and mRFP dots are red.

Autophagy, or self-eating, is a self-protection strategy, which can maintain normal intracellular homeostasis through removing misfolded proteins, cleaning impaired organelles [18–20]. Autophagy is a vital movement which contains three main steps: formation of autophagosomes, the fusion of autophagosomes with lysosomes and degradation [18, 21, 22]. According to the results that autophagosomes are growing in a time-dependent manner shown by the TEM, given that autophagy is not a static but a gradual and dynamic process, we performed autophagic flux to assess it accurately. We use mRFP-GFP-LC3 Puncta Formation Assays to evaluate the level of autophagic flux, as it can discriminate between autophagosomes and autolysosomes [23, 24]. GFP fluorescence signal is significantly quenched by the low pH while RFP fluorescence signal remain fluorescent in acidic environment [23, 25]. Therefore, autophagosomes and autolysosomes appear as yellow and red puncta, respectively. Thus, autophagic flux can be measured by exploiting the two different fluorescent signatures (Figure 5). The results suggested that autophagosomes were growing with the increasing concentration of CONPs, which were consistent with the findings of TEM (Figure 3).

**Figure 6:**
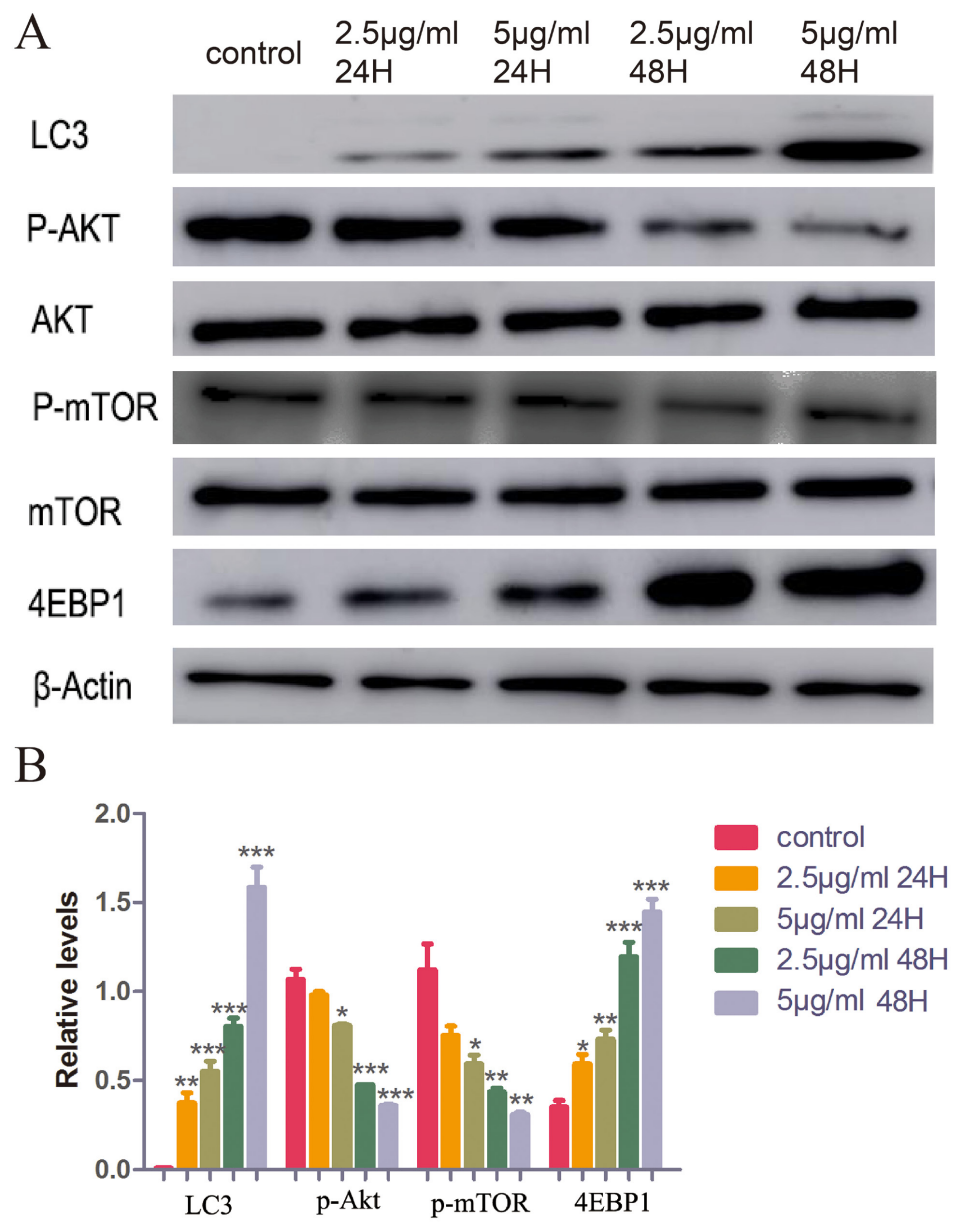
Western blot assay of LC3, AKT, p-AKT, mTOR, p-mTOR, 4EBP1 and β-actin (**A**) The conversion of LC3-I to LC3-II and the expression of AKT, p-AKT, mTOR, p-mTOR and 4EBP1 were detected using specific antibodies while β-actin served as loading control. (**B**) Below a graphical representation of the combined data of three individual experiments is presented. LC3 and 4EBP1 expression levels were standardized using the respective expression of actin. P-mTOR expression levels were standardized using the respective expression of mTOR. P-Akt expression levels were standardized using the respective expression of AKT. *,P < 0.05; **, P < 0.01; ***, P < 0.001 relative to control.

Western blot was performed to further analyze the potential targets. It was found that LC3, the marker of autophagy [26, 27], increased in response to treatment with CONPs in a dose- and time-dependent manner. It was also found that CONPs could decrease the expression of p-AKT, p-mTOR, and increase the expression of 4EBP1, which are crucial in the development of cancer [28–30] (Figure 6).

## DISCUSSION

Cuprous oxide nanoparticle, a kind of nanomedicine, has broad prospects in anti-tumor therapy. Our group discovered that CONPs could inhibit melanoma cell invasion and metastasis and show little hepatotoxicity and renal toxicity.

In the present research, we studied the effects of CONPs on cervical cancer cell lines. The data indicated that CONPs could inhibit the cell proliferation in a concentration- and time-dependent manner which may be concerned with that CONPs can induce apoptosis and block cell cycle at G1/G0 phase. The study with subcutaneous cervical carcinoma mice model indicated that CONPs had an ideal anti-tumor capacity compare with cisplatin.

Autophagic dysfunction is associated with an astonishing number of disease and pathophysiological, such as cancer, neurodegeneration, ageing [18, 31, 32]. According to the TEM and autophagy flux, CONPs could induce the formation of autophagosomes in a time- and concentration-dependent manner. The AKT/mTOR signaling pathway is closely related to the activity of autophagy [33–35]. So, we performed Western blot to detect the effects of CONPs on AKT/ mTOR pathway. The data showed that CONPs could decrease the expression of P-AKT, P-mTOR, strongly suggesting that CONPs could induce autophagy through AKT/mTOR pathway.

As is commonly known, cervical cancer is a “poor” cancer, even with a widespread application of vaccine of cervical cancer, the incidence of cervical cancer in the development country is still staying at a high level. Currently the treatment of cervical cancer relies on surgery and chemotherapy. Unfortunately, chemotherapy has many serious side effects, such as weight loss, hepatotoxicity and renal toxicity, which greatly limit the application of chemotherapy and the therapeutic outcome. Therefore, it is of considerable importance and significance to find a medicinal product that is effective in killing cancer cells, low-cost, and has few side effects, thereby ultimately having the potential of clinical application. Our team found that the synthesis of CONPs is convenient and inexpensive; according to our previous study, CONPs have little hepatotoxicity and renal toxicity. Compare with cisplatin, CONPs have a comparable inhibitory effect on tumor growth without the side effect of weight loss. Our data provide some initial evidence that CONPs might be of therapeutic potential in the treatment of cervical cancer.

## MATERIALS AND METHODS

### Synthesis method

The synthetic method of CONPs are introduced in our previous study. All chemicals used in the experiment were of analytical grade. The synthetic method of CONPs are as follows: First, 0.35 mL of 0.1 M CuSO4 (aq) and 3 mL of 0.1 M cetyltrimethylammonium bromide (CTAB) (aq) were added into a test tube, the tube was shaken vigorously by a vortex mixer. The mixture turned red-brown rapidly when the 10 mL of fresh, cold 0.04 M NaBH_4_ (aq) was added into it. The test tube was in the incubation at 26°C–28°C until the mixture turned into bright yellow for about 18 hours. The solution was centrifuged at 26°C for 15-20 minutes at 12,000 rpm and washed with ethanol and deionized water for several times. After removing the supernatant, the vacuum dry method is applicable for the product. After about 15 hours, the dried product turned into black powder and was stored in a 4°C refrigerator [13, 14].

### Cell culture

The HeLa cells, SiHa cells, MS751 cells, and Caski cells used in this study were purchased from the Cell Bank of Typical Culture Collection (Chinese Academy of Sciences, Shanghai, China). All the cells were grown in Dulbecco’s modified Eagle’s medium (DMEM, Gibco™, Invitrogen Corp, Carlsbad, CA) supplemented with 10%FBS, 100 µg/mL streptomycin sulfate, 40 µg/mL gentamicin, 100 U/mL penicillin. The cells were seeded in culture dishes and placed in the humidified incubator at 37°C with 5% CO_2_.

### Cell proliferation assay

In this assay, the Cell Counting Kit-8 (CCK-8) assay (Dojindo Laboratories, Kumamoto, Japan) was used to access the effects of CONPs on proliferation ability of the cells. Cells in exponential phase of growth were implanted at a concentration of 4000 cells/well in 96-well plates. After 24 hours incubating, the DMEM was replaced by fresh medium containing various concentrations of CONPs. After 24 hours, 48 hours, 72 hours, cell viability was measured by CCK-8 assay. The absorbance was detected by a microplate reader (Tiilitie, Finland) at 450 nm. The average absorbance for each concentration of CONPs were calculated from six wells.

### Cell migration and invasion assays

For cell migration assay, 1 × 10^4^ MS751 cells with different concentrations of CONPs were added onto the upper chamber in 1% FBS medium and full growth medium loaded in the lower chamber. 24 hours later, the cells were washed and fixed in 4% paraformaldehyde for 20 min. Then the noninvasive cells were removed from the upper chamber with a cotton swab. The membranes were stained with crystal violet for about 15 min. The number of cells migrated through the membrane was counted. The experiments were repeated 3 times. Cell counts were performed on five random fields in each well. Cell invasion assay was performed following the procedure similar the cell migration assay except that the membrane was pre-coated with Matrigel.

### Apoptosis assay

Apoptosis assay was performed using an Annexin V-FITC/PI Apoptosis Detection Kit (Multi Sciences (Lianke) Biotech Co., Ltd., Hangzhou, China) by flow cytometry according to the protocol provided. Briefly, HeLa cells, SiHa cells, MS751 cells, and Caski cells were incubated with various concentration of CONPs for 48 hours after cells were adherent to the bottom. Then the cells were centrifugated and stained with Annexin V and PI from light. After 15 min, the samples were analyzed immediately by flow cytometry (Accuri C6; BD Biosciences). Each experiment was carried out for at least three times.

### Cell cycle assay

Cell cycle assay was assessed by flow cytometry using a cell cycle staining Kit (Multi Sciences (Lianke) Biotech Co., Ltd., Hangzhou, China) according to the protocol provided. Briefly, HeLa cells, SiHa cells, MS751 cells, and Caski cells were incubated with various concentration of CONPs for 48 hours after cells were adherent to the bottom. Then the cells were fixed with 75% ethanol and resuspended in reagent A. After half an hour, the samples were analyzed by flow cytometry (Accuri C6; BD Biosciences). Each experiment was carried out for at least three times.

### Analysis of cell morphology

Cells morphology of HeLa cells and MS751 cells treated with various concentration of CONPs for 48 hours were assessed by electron microscopy (IX81, Olympus).

### Anti-tumor property assay of nude mice with subcutaneous cervical carcinoma

2 × 10^6^ MS751 cells were inoculated subcutaneously in nude mice. Once the mean tumor diameter reached 6 mm these mice were divided randomly into 4 groups(n=6/group), 4 mg/kg CONPs or 2 mg/kg DDP or 4 mg/kg CONPs + 2 mg/kg DDP. All the mice were sacrificed on day 20 after the first treatment. The volume of the tumors was measured with Vernier caliper and the tumor growth suppression rate was calculated.

### Transmission electron microscopy (TEM) assay

The MS751 cells were incubated with CONPs for 6 hour and 9 hour. Cells were dehydrated with increasing concentrations of ethanol after incubating with formaldehyde. At last, cells were stained in uranyl acetate, embedded in Epon, made into ultrathin section and observed with transmission electron microscope (Hitachi, Tokyo, Japan).

### Mitochondrial membrane potential

Mitochondrial membrane potential was measured by confocal laser microscopy and flow cytometry using the JC-1 Mitochondrial membrane Potential Assay Kit(C2006, Beyotime Biotechnology, Shanghai, China ) . Cells were grown on glass coverslips and treated with CONPs in various concentrations for 48 h. After incubating with JC-1 for 20 min, the cells were washed with staining buffer and detected by confocal laser microscopy (SP5, Leica) or flow cytometry (Accuri C6; BD Biosciences) immediately.

### Autophagy flux analysis

Cells infected with the mRFP-GFP-LC3 adenoviral particles (HANBIO Biotechnology Co., Ltd, Shanghai, China) were treated with various concentration of CONPs for 72 h. Imaging was performed on confocal laser microscopy (SP5, Leica). Settings during image acquisition were kept constant.

### Western blotting analysis

The protein was extracted from MS751 cells treated with CONPs by RIPA buffer containing proteinase inhibitor cocktail and phosphatase inhibitor cocktail. Proteins were separated by 10% or 12% SDS/PAGE gels and transferred to polyvinylidene fluoride membranes. After blocking with BSA for 2 hours, the blots were incubated with primary antibodies at 4 °C overnight. Then the blots were incubated with secondary antibodies for 2 hours after washing with TBST for three times. After washing three times, the blots were incubated with Super Signal West Pico chemiluminescent substrate and analyzed using the GeneGnome HR Image Capture System.

## Abbreviations

DDP: Cisplatin
